# Inhibition of Bovine Enterovirus Infection by Magnolol via Modulating the Gut Microbiota in Mice

**DOI:** 10.3390/v17060750

**Published:** 2025-05-24

**Authors:** Junying Hu, Qun Zhang, Dan Liu, Xuyuan Cui, Qianying Wang, Wenjie Gong, Xinping Wang

**Affiliations:** 1 State Key Laboratory for Diagnosis and Treatment of Severe Zoonotic Infectious Diseases, Key Laboratory for Zoonosis Research of the Ministry of Education, Institute of Zoonosis, College of Veterinary Medicine, Jilin University, Changchun 130012, China; jyhu19@mails.jlu.edu.cn (J.H.); zhangqun21@mails.jlu.edu.cn (Q.Z.); cuixy22@mails.jlu.edu.cn (X.C.); qianying22@mails.jlu.edu.cn (Q.W.); 2 National Key Laboratory of Intelligent Tracking and Forecasting for Infectious Diseases (NITFID), NHC Key Laboratory for Medical Virology and Viral Diseases, National Institute for Viral Disease Control and Prevention, Chinese Center for Disease Control and Prevention, Beijing 102206, China; liudanxx361612349@126.com

**Keywords:** magnolol, bovine enterovirus, gut microbiota, mouse model

## Abstract

Bovine enterovirus (BEV) infection is one of the important infectious diseases that cause digestive and respiratory symptoms in cattle, posing a significant threat to the cattle industry. Currently, no vaccines or therapeutic drugs are available for this disease. In our study, we utilized a mouse model to investigate the effects of BEV infection on the gut microbiota and examine the therapeutic potential of magnolol (Mag), a polyphenolic bioactive substance, in terms of BEV infection. BEV infection significantly altered the microbiota composition, where the abundance of some beneficial bacteria, such as *Lactobacillaceae* and *Lactobacillus*, was markedly reduced. Mag effectively inhibited BEV infection in vivo. Upon BEV infection, Mag treatment reduced the α-diversity of the microbiota, with statistically significant differences on day 3 post-infection compared to the Mag-untreated group. More interestingly, Mag treatment significantly reversed the effect of BEV infection on the *Lactobacillaceae* and *Lactobacillus* abundance, indicating that Mag positively regulates beneficial bacteria. The fecal microbiota transplantation (FMT) experiment demonstrated that feces from Mag-treated mice significantly decreased the virus loads in the small intestine samples of BEV-infected mice. These findings demonstrate the interaction between BEV infection and the gut microbiota and highlight the important regulatory role of the gut microbiota in Mag’s anti-BEV effects, opening up a new avenue for preventing and controlling BEV infection via targeted modulation of the gut microbiota.

## 1. Introduction

Bovine enterovirus (BEV) infection is an emerging animal epidemic disease in China that causes severe losses for the cattle industry. As the causative agent of this disease, BEV belongs to the genus *Enterovirus* within the family *Picornaviridae* [1] and is categorized into *Enterovirus eibovi* and *Enterovirus fitauri* [2]. Since its initial discovery in cattle in the late 1950s, infection caused by this virus has spread widely across the globe [3,4,5,6,7,8]. Epidemiological investigations showed that BEV infection typically leads to respiratory and digestive diseases in hosts and can spread rapidly through the fecal–oral route, posing a severe threat to the farming industry [3,6,9]. Therefore, it is of great importance for us to prevent and control BEV infection.

The gut microbiota fulfills various physiological and biochemical functions in animals and mediates the pathogenesis of diseases, including inflammation, cancers, metabolic diseases, neurological diseases, and viral infections [10,11,12,13,14]. Additionally, studies have demonstrated that certain drugs can prevent and control viral diseases by targeting the gut microbiota. Matrine targets *Lactobacillus acidophilus* to suppress porcine circovirus type 2 infection [15]. Phlorizin modulates the gut microbiota to inhibit bovine viral diarrhea virus infection [16]. Chinese herbal formulations like Fei-Yan-Qing-Hua decoction and Ruhao Dashi granules prevent influenza virus infections by altering the gut microbiota [17,18]. These studies demonstrated that the gut microbiota plays a pivotal role in the host’s antiviral defense.

Magnolol (Mag), a natural polyphenol isolated from the Magnoliaceae plant *Magnolia officinalis*, exhibits anti-inflammatory, anti-tumor, and antioxidant functions [19,20,21]. Mag also has antiviral effects, as demonstrated in the inhibition of enterovirus 71, human norovirus surrogates, porcine epidemic diarrhea virus, hepatitis B virus, and grass carp reovirus [22,23,24,25,26]. Although the antiviral effects of Mag on given viral infection have been confirmed, its antiviral effect on BEV is unknown.

In our research, we investigated the interaction of BEV infection with the gut microbiota and the effect of Mag on BEV infection through a mouse model. The results showed that BEV infection induced a marked disturbance in the gut microbiota and Mag-mediated gut microbiota modulation effectively inhibited BEV infection. These discoveries provide insights into the antiviral effect of Mag and its underlying mechanism, laying a foundation for formulating new strategies to prevent and control BEV infection.

## 2. Materials and Methods

### 2.1. Virus and Mice

Bovine enterovirus HY12 strain (GenBank accession no. KF748290) was isolated by our laboratory [6]. Three-week-old female ICR mice were obtained from Changsheng (Benxi, China) [27,28]. This research was conducted in accordance with the regulation (JLU-20150226) of the Institutional Animal Care and Use Committee (IACUC) of Jilin University.

### 2.2. Reagents

Magnolol (≥98% HPLC) was acquired from Must (Chengdu, China). The rabbit anti-BEV VP1 polyclonal antibody was generated in our laboratory. The rabbit anti-GAPDH pAb was acquired from ABclonal (Wuhan, China). The goat anti-rabbit IgG conjugated with horseradish peroxidase (HRP) antibody was acquired from Immunoway (San Jose, CA, USA).

### 2.3. Experimental Design

To prove the dynamic changes in the gut microbiota induced by BEV, six mice were each administered an intraperitoneal (IP) injection of 0.2 mL of culture medium containing 2 × 10^8^ TCID_50_ of the virus. Fresh fecal specimens were collected for metagenomic sequencing of the gut microbiota on day 1 and day 3 post-infection. The mice feces before BEV infection was collected and used as a control (day 0).

For investigating the impact of magnolol on the microbiota in BEV-infected mice, 20 mice were randomly assigned to either the magnolol treatment group (Mag) or the untreated group (Con). Magnolol was placed in a sterile solvent containing 5% DMSO and 2% Tween-80. Seven days prior to BEV infection, the Mag group received daily doses of magnolol (100 mg/kg) via oral gavage, whereas the Con group received an equivalent volume of sterile solvent containing 5% DMSO and 2% Tween-80. The safe dosage and administration time for magnolol were referenced from previous studies [29,30,31,32,33,34]. The magnolol treatment continued throughout the course of the BEV infection. On day 1 post-infection (1 dpi), fecal specimens were collected from 5 mice per group for metagenomic sequencing of the gut microbiota. Simultaneously, small intestine samples were collected from the mice for pathogen detection. The same procedure was performed on day 3 post-infection (3 dpi).

To clarify the impact of the gut microbiota on magnolol’s anti-BEV effect, 10 mice were assigned to two groups. Five mice in group 1 received magnolol treatment, while another 5 mice in group 2 were given just the sterile solvent. After 7 days of treatment, the feces samples from both groups were collected daily and used as fecal microbiota transplantation (FMT) donors until the end of the BEV infection. For the FMT experiments [15,16], 10 mice were assigned to two groups, named Mag-FMT and Con-FMT, respectively. The mice in the Mag-FMT group were given the fecal treatment solution collected from the mice treated with Mag, while the mice in Con-FMT group were given the fecal treatment solution from the mice treated with the sterile solvent. Three days post-FMT, all the mice were infected with BEV at a dose of 2 × 10^8^ TCID_50_ via intraperitoneal (IP) injection. Three days post-infection, the mice in both groups were euthanized. The small intestine samples were collected for BEV detection.

### 2.4. Gut Microbiota Analysis

The total DNA was extracted from the feces and then amplified via PCR. The resulting fragments were purified, quantified, and homogenized to create sequencing libraries. Once the libraries passed quality control, sequencing was performed. The operational taxonomic unit (OTU) was defined as sequences with over 97% similarity. We applied α-diversity to evaluate the species diversity and richness, and we employed β-diversity to assess the species variations across samples [14].

### 2.5. Quantitative Real-Time Polymerase Chain Reaction

The total RNA was isolated from the specimens using RNAiso Plus (Takara, Japan), followed by cDNA synthesis with the ABclonal (China) reverse transcription system. Quantitative PCR (qPCR) amplification of different genes was conducted with 2 × SYBR Green qPCR Mix (ABclonal, China). The calculation method of 2^−ΔΔCt^ was employed for processing the qPCR data [35]. The primer sequences for this research are provided in Table 1.

### 2.6. Western Blot

The total protein was harvested from the lysates of the mouse tissue specimens and quantified with BCA Protein Quantitative Kit (Beijing, TransGen, China). SDS-PAGE was performed to separate the proteins, which were then transferred onto the PVDF membrane. Following the 2 h incubation with 5% nonfat milk for blocking, the membrane was treated with the primary antibodies against VP1 or GAPDH. Subsequently, it was probed with the secondary antibody and visualized using a Western blot detection system [16].

### 2.7. Statistical Analysis

This study used GraphPad Prism 9 software for the statistical analysis. The Shapiro–Wilk test was applied to assess the normality of the data. For normally distributed data, the unpaired *t*-test or Welch’s *t*-test was used to compare the differences between two groups. For comparisons among three groups, the one-way ANOVA with Tukey’s multiple comparisons test or the Brown–Forsythe and Welch ANOVA with Dunnett’s T3 multiple comparisons test was applied. All the data were presented as the mean ± standard deviation (SD). For data with a non-normal distribution, the Mann–Whitney test was used for the two-group comparisons, and the Kruskal–Wallis test with Dunn’s multiple comparisons test was applied for comparisons among three groups [36]. * *p* < 0.05 or ** *p* < 0.01 indicated significance.

## 3. Results

### 3.1. Magnolol Inhibits BEV Replication

**Figure 1:**
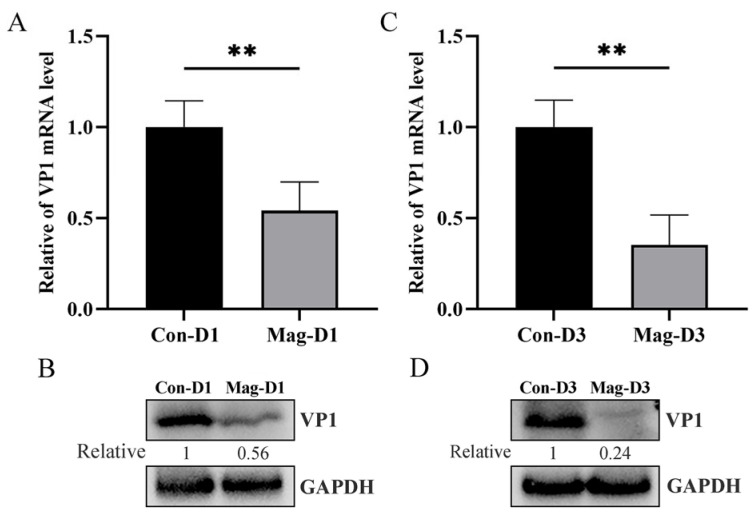
Magnolol inhibits BEV replication in mice. (**A**) The BEV RNA level in the small intestine at 1 dpi was determined using RT-qPCR. Unpaired *t*-test, *p* = 0.005. (**B**) The level of BEV VP1 protein in the small intestine at 1 dpi was measured by Western blot. (**C**) The BEV RNA level in the small intestine at 3 dpi was determined using RT-qPCR. Unpaired *t*-test, *p* = 0.001. (**D**) The level of BEV VP1 protein in the small intestine at 3 dpi was measured by Western blot. ** *p* < 0.01.

To explore the impact of Mag on BEV replication, BEV-infected mice were treated with or without Mag. The viral loads were measured in the small intestine of the mice at 1 dpi and 3 dpi. Compared with the Mag-untreated group, the BEV RNA levels in the small intestine of the mice treated with Mag were significantly reduced (Figure 1A,C), and the BEV protein levels were correspondingly decreased (Figure 1B,D), suggesting that Mag inhibits BEV replication in vivo.

### 3.2. Richness and Diversity of the Microbiota After BEV Infection

**Figure 2:**
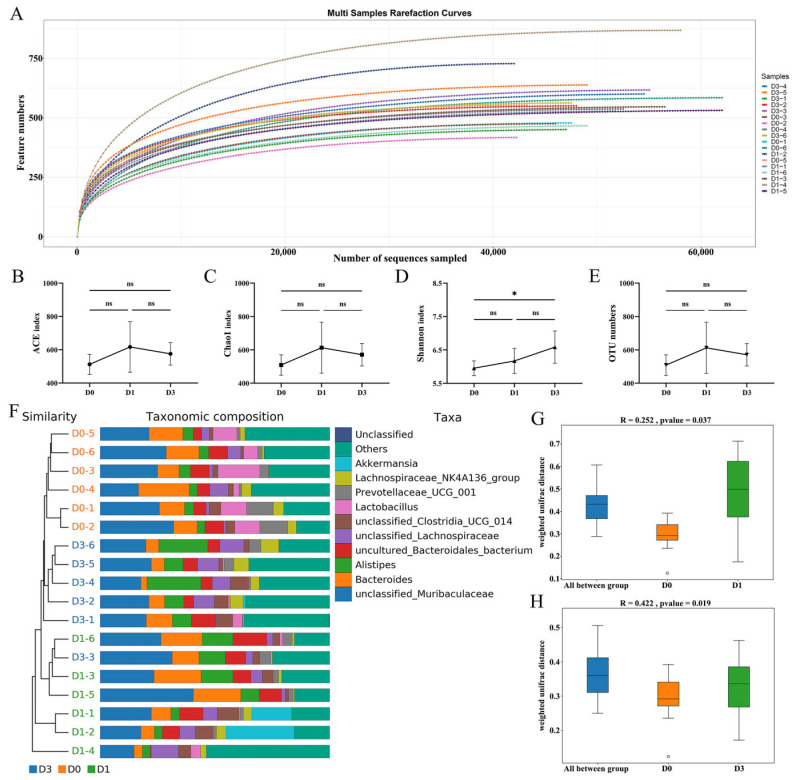
Variations in the gut microbiota caused by BEV infection at 1 dpi and 3 dpi. (**A**) Rarefaction curves of the D0 (Day 0, before BEV infection), D1 (Day 1, post-infection), and D3 (Day 3, post-infection) groups. (**B**) ACE index. One-way ANOVA and Tukey’s multiple comparisons test. (**C**) Chao1 index. One-way ANOVA and Tukey’s multiple comparisons test. (**D**) Shannon index. One-way ANOVA and Tukey’s multiple comparisons test. (**E**) OTU numbers. One-way ANOVA and Tukey’s multiple comparisons test. (**F**) Bar chart of the taxonomic composition of gut microbiota in the D0, D1, and D3 groups. (**G**) ANOSIM analysis to assess the variation in community structure between the D0 and D1 groups. (**H**) ANOSIM analysis to evaluate the alteration in community structure between the D0 and D3 groups. * *p* < 0.05; ns, not significant.

For investigating the alterations in the microbiota resulting from BEV infection, fecal specimens from the experimental mice were collected on days 0, 1, and 3 for microbiota sequencing. The rarefaction curves demonstrated that the sequencing depth was adequate across all the samples (Figure 2A). Compared to the D0 group, no significant variations were observed in the ACE (D0 vs. D1, *p* = 0.212; D0 vs. D3, *p* = 0.546) and Chao1 (D0 vs. D1, *p* = 0.219; D0 vs. D3, *p* = 0.559) indices, the measurement of species richness, across the different time points after BEV infection (Figure 2B,C). However, the Shannon index, reflecting the species diversity, increased significantly at 3 dpi (D0 vs. D3, *p* = 0.027) (Figure 2D). As illustrated in Figure 2E, no significant alterations in the OTU numbers were observed following BEV infection (D0 vs. D1, *p* = 0.222; D0 vs. D3, *p* = 0.562; D1 vs. D3, *p* = 0.768). Additionally, microbial composition analysis revealed that the species compositions of the D0, D1, and D3 groups were relatively clustered (Figure 2F). ANOSIM analysis showed notable variations in the composition of the microbiota between the D0 and D1 groups (*p* = 0.037), as well as between the D0 and D3 groups (*p* = 0.019) (Figure 2G,H).

### 3.3. BEV Infection Affects the Composition of the Gut Microbiota at the Phylum and Family Levels

**Figure 3:**
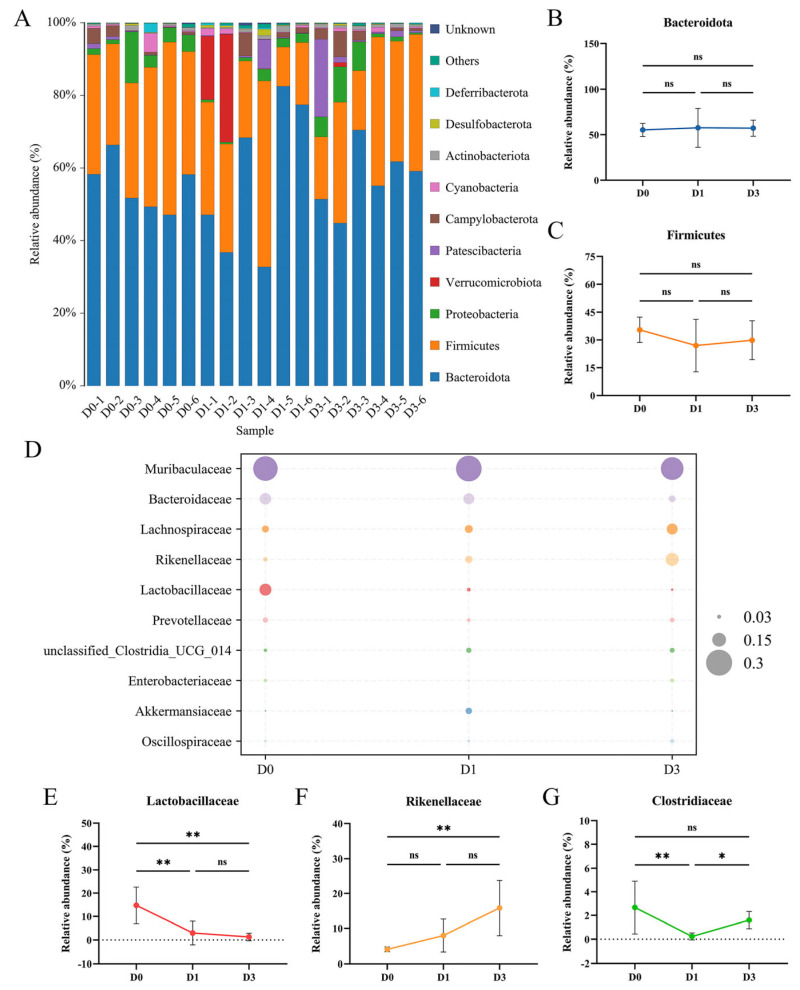
Impact of BEV infection on the microbial communities at the phylum and family levels after 1 and 3 days. (**A**) Bar chart of the microbiota at the phylum level. (**B**) Relative abundance of Bacteroidota. Brown–Forsythe and Welch ANOVA tests with Dunnett’s T3 multiple comparisons test. (**C**) Relative abundance of Firmicutes. One-way ANOVA and Tukey’s multiple comparisons test. (**D**) Bubble chart of the microbial community at the family level. (**E**) Relative abundance of *Lactobacillaceae*. One-way ANOVA and Tukey’s multiple comparisons test. (**F**) Relative abundance of *Rikenellaceae*. One-way ANOVA and Tukey’s multiple comparisons test. (**G**) Relative abundance of *Clostridiaceae*. Kruskal–Wallis test and Dunn’s multiple comparisons test. * *p* < 0.05; ** *p* < 0.01; ns, not significant.

Analyses of the microbial composition at the phylum level revealed 10 major phyla across all the samples, including Bacteroidota, Firmicutes, Proteobacteria, Verrucomicrobiota, Patescibacteria, Campylobacterota, Cyanobacteria, Actinobacteriota, Desulfobacterota, and Deferribacterota. Among the 10 phyla, Bacteroidota and Firmicutes were the most abundant (Figure 3A). No significant alterations in the abundance of Bacteroidota (D0 vs. D1, *p* = 0.991; D0 vs. D3, *p* = 0.964; D1 vs. D3, *p* > 0.999) and Firmicutes (D0 vs. D1, *p* = 0.392; D0 vs. D3, *p* = 0.655; D1 vs. D3, *p* = 0.891) between the D0, D1, and D3 groups were observed (Figure 3B,C). Microbial composition analysis indicated that *Muribaculaceae*, *Bacteroidaceae*, *Lachnospiraceae*, *Rikenellaceae*, and *Lactobacillaceae* were the five most abundant families (Figure 3D). Furthermore, after BEV infection, the abundance of certain microbial families associated with health changed. Compared to the D0 group, BEV infection resulted in a continuous reduction in the abundance of *Lactobacillaceae* at 1 dpi (D0 vs. D1, *p* = 0.005) and 3 dpi (D0 vs. D3, *p* = 0.002), with statistically significant reductions observed at both time points (Figure 3E). Conversely, the relative abundance of *Rikenellaceae* showed a continuous increase, with statistical significance observed only at 3 dpi (D0 vs. D3, *p* = 0.004) (Figure 3F). Additionally, low-abundance *Clostridiaceae* exhibited a significant decrease at 1 dpi (D0 vs. D1, *p* = 0.006) (Figure 3G).

### 3.4. BEV Infection Changes the Composition of the Gut Microbiota at the Genus Level

**Figure 4:**
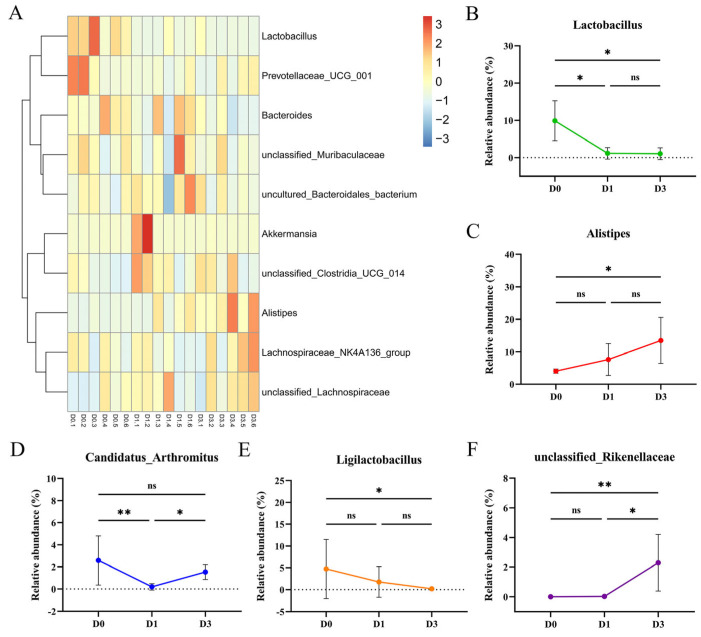
Effect of BEV infection on the microbiota at the genus level after 1 or 3 days. (**A**) Heatmap of the microbial community at the genus level. (**B**) Relative abundance of *Lactobacillus*. Kruskal–Wallis test and Dunn’s multiple comparisons test. (**C**) Relative abundance of *Alistipes*. Kruskal–Wallis test and Dunn’s multiple comparisons test. (**D**) Relative abundance of *Candidatus_Arthromitus*. Kruskal–Wallis test and Dunn’s multiple comparisons test. (**E**) Relative abundance of *Ligilactobacillus*. Kruskal–Wallis test and Dunn’s multiple comparisons test. (**F**) Relative abundance of *unclassified_Rikenellaceae*. Kruskal–Wallis test and Dunn’s multiple comparisons test. * *p* < 0.05; ** *p* < 0.01; ns, not significant.

Genus-level analysis further demonstrated the changes in the microbial composition following BEV infection (Figure 4A). Notably, some beneficial bacteria showed significant alterations, such as Lactobacillus and Ligilactobacillus. At 1 dpi, the relative abundance of Lactobacillus (D0 vs. D1, *p* = 0.024) and Candidatus_Arthromitus (D0 vs. D1, *p* = 0.006) was markedly decreased compared to the uninfected D0 group (Figure 4B,D). At 3 dpi, the relative abundance of Lactobacillus continued to decrease (D0 vs. D3, *p* = 0.013) (Figure 4B), while Ligilactobacillus (D0 vs. D3, *p* = 0.015) also showed a significant decline compared to the D0 group (Figure 4E). Additionally, the relative abundance of Alistipes (D0 vs. D3, *p* = 0.018) and unclassified_Rikenellaceae (D0 vs. D3, *p* = 0.001) increased significantly at 3 dpi (Figure 4C,F).

### 3.5. Magnolol Treatment Alters the Diversity and Richness of the Microbiota

**Figure 5:**
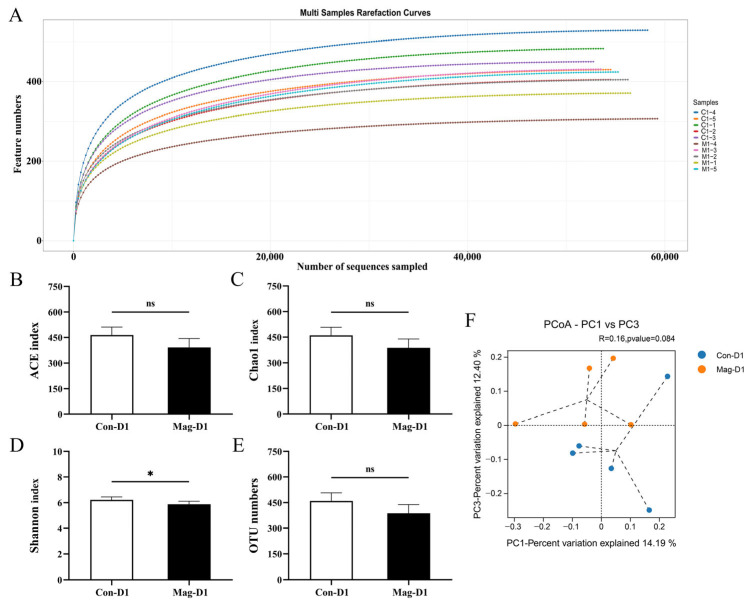
Influence of Mag treatment on the gut microbiota of BEV-infected mice at 1 dpi. (**A**) Rarefaction curves of the Con-D1 group and Mag-D1 group. (**B**) ACE index. Unpaired *t*-test. (**C**) Chao1 index. Unpaired *t*-test. (**D**) Shannon index. Unpaired *t*-test. (**E**) OTU numbers. Unpaired *t*-test. (**F**) PCoA generated using the unweighted UniFrac distance matrices. * *p* < 0.05; ns, not significant.

**Figure 6:**
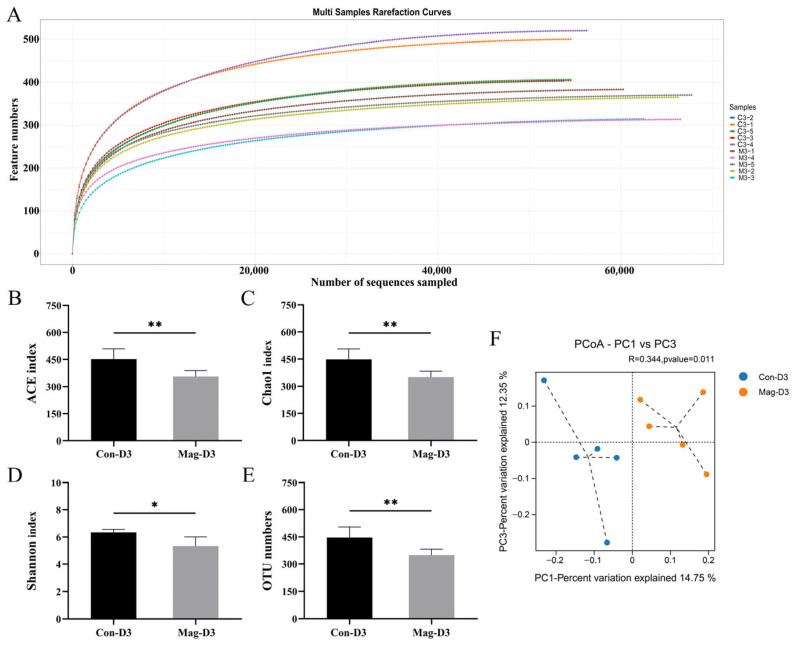
Impact of Mag treatment on the gut microbiota of BEV-infected mice at 3 dpi. (**A**) Rarefaction curves of the Con-D3 group and Mag-D3 group. (**B**) ACE index. Mann–Whitney test. (**C**) Chao1 index. Mann–Whitney test. (**D**) Shannon index. Unpaired *t*-test. (**E**) OTU numbers. Mann–Whitney test. (**F**) PCoA generated using the unweighted UniFrac distance matrices. * *p* < 0.05; ** *p* < 0.01.

For investigating the effects of magnolol treatment on the microbial community in BEV-infected mice, fecal samples were collected from the Mag-untreated groups (Con-D1 and Con-D3) and Mag treatment groups (Mag-D1 and Mag-D3) at 1 dpi and 3 dpi for microbiota analysis. The sequencing depth for all the fecal specimens was adequate (Figure 5A and Figure 6A). At 1 dpi, the Mag-treated group (Mag-D1) exhibited lower α-diversity of the microbial community compared to the Mag-untreated group (Con-D1) (Figure 5B–D). Simultaneously, no notable alteration in the OTU numbers was observed with or without Mag treatment at 1 dpi (*p* = 0.051) (Figure 5E). By the third day of infection, the α-diversity of the microbiota in the Mag-treated group (Mag-D3) remained at reduced levels compared to the untreated group (Con-D3), with significant differences observed in the ACE (*p* = 0.008), Chao1 (*p* = 0.008), and Shannon indices (*p* = 0.014) (Figure 6B–D). Meanwhile, a significant variation in the OTU numbers was also observed with or without Mag treatment at 3 dpi (*p* = 0.008) (Figure 6E). Furthermore, β-diversity analysis revealed that the microbial communities formed distinct clusters between the Con-D1 and Mag-D1 groups, as well as between the Con-D3 and Mag-D3 groups (Figure 5F and Figure 6F).

### 3.6. Magnolol Treatment Affects the Microbiota Composition at the Phylum and Family Levels

**Figure 7:**
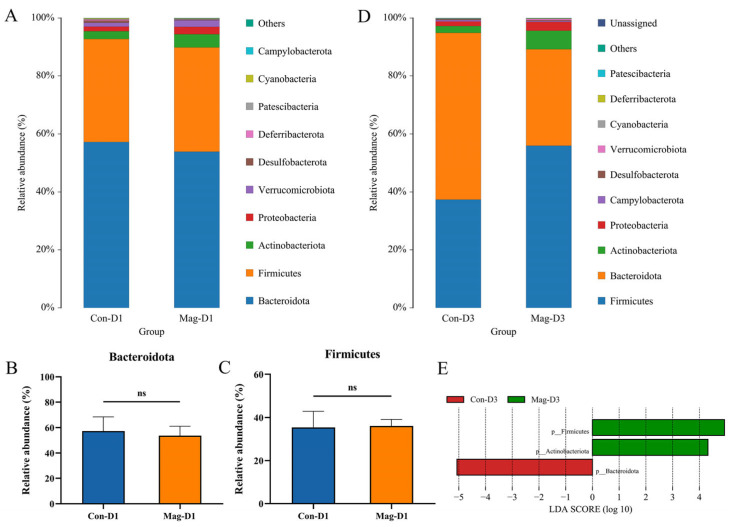
Influence of Mag treatment on the microbiota at the phylum level after 1 or 3 days of BEV infection. (**A**) Bar chart showing the microbiota at the phylum level following 1 day of BEV infection. (**B**) Relative abundance of Bacteroidota at 1 dpi. Unpaired *t*-test. (**C**) Relative abundance of Firmicutes at 1 dpi. Unpaired *t*-test. (**D**) Bar chart showing the microbiota at the phylum level following 3 days of BEV infection. (**E**) LEfSe analysis at the phylum level after 3 days of BEV infection (taxa with LDA score > 4). ns, not significant.

To further probe the effects of magnolol treatment on the microbial community composition in BEV-infected mice, the bacterial species and their abundance at the phylum level were analyzed. Regardless of whether it was day 1 or day 3 post-infection, Bacteroidota and Firmicutes consistently dominated as the two most abundant phyla in the fecal samples (Figure 7A,D). Although there were no significant variations in the relative abundance of Bacteroidota (*p* = 0.557) and Firmicutes (*p* = 0.849) between the Con-D1 and Mag-D1 groups at 1 dpi (Figure 7B,C), notable variations in the abundance of these two phyla were observed on day 3. At 3 dpi, the abundance of Firmicutes and Actinobacteriota was significantly increased following Mag treatment, whereas that of Bacteroidota was markedly decreased (Figure 7E).

**Figure 8:**
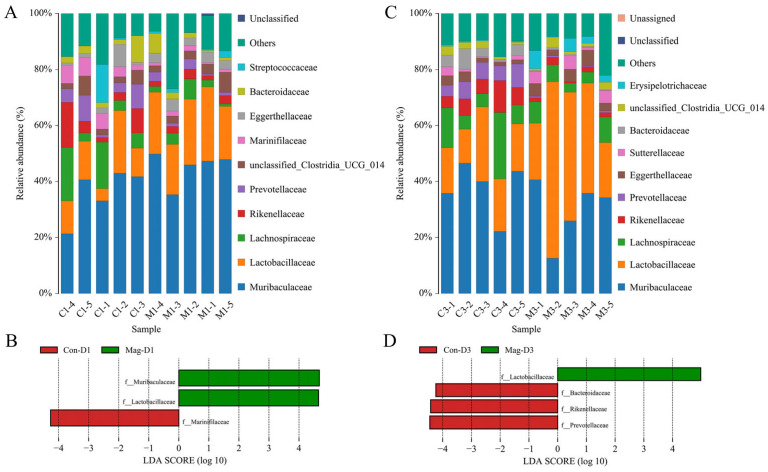
Influence of Mag treatment on the microbial communities at the family level after 1 and 3 days of BEV infection. (**A**) Bar chart illustrating the microbiota at the family level following 1 day of BEV infection. (**B**) LEfSe analysis at the family level after 1 day of BEV infection (taxa with LDA score > 4). (**C**) Bar chart illustrating the microbial community at the family level following 3 days of BEV infection. (**D**) LEfSe analysis at the family level after 3 days of BEV infection (taxa with LDA score > 4).

Simultaneously, the bacterial species and their abundance at the family level were analyzed. Changes in several bacterial families were revealed after Mag treatment (Figure 8A,C). At 1 dpi, Mag treatment significantly increased the relative abundance of Muribaculaceae and Lactobacillaceae, whereas it significantly decreased the abundance of Marinifilaceae (Figure 8B). At 3 dpi, the abundance of Lactobacillaceae persisted at a significantly elevated level following Mag treatment, whereas that of Bacteroidaceae, Rikenellaceae, and Prevotellaceae exhibited significant reductions (Figure 8D).

### 3.7. Microbiota Composition at the Genus Level Following Magnolol Treatment

**Figure 9:**
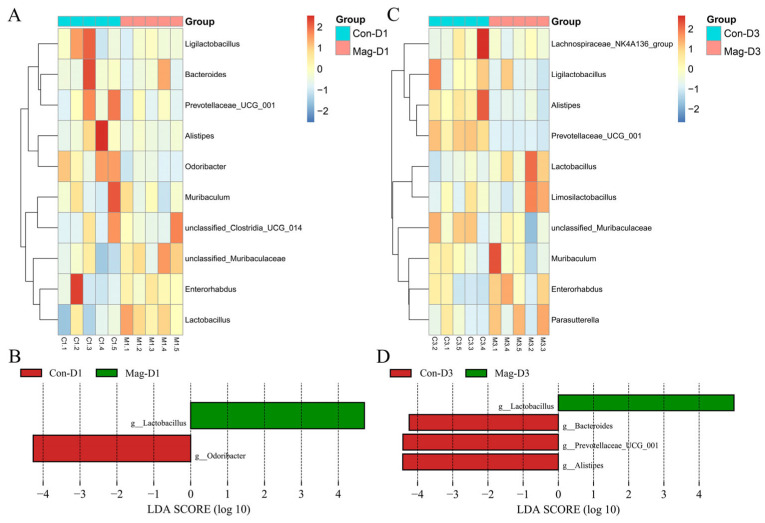
Influence of Mag treatment on the microbial communities at the genus level after 1 or 3 days of BEV infection. (**A**) Heatmap showing the microbial community at the genus level following 1 day of BEV infection. (**B**) Analysis of the LEfSe at the genus level after 1 day of BEV infection (taxa with LDA score > 4). (**C**) Heatmap showing the microbial community at the genus level following 3 days of BEV infection. (**D**) Analysis of the LEfSe at the genus level after 3 days of BEV infection (taxa with LDA score > 4).

Further analysis of the variations in the microbiota at the genus level was performed using heatmaps (Figure 9A,C). Notably, the relative abundance of the probiotic Lactobacillus was markedly increased at both 1 dpi and 3 dpi following Mag treatment, indicating a positive regulatory effect of magnolol on probiotic bacteria (Figure 9B,D). Additionally, Mag treatment reduced the relative abundance of Odoribacter at 1 dpi (Figure 9B) and decreased the abundance of Bacteroides, Prevotellaceae_UCG_001, and Alistipes at 3 dpi (Figure 9D).

### 3.8. Functional Alterations in the Microbiota Induced by Magnolol Treatment

**Figure 10:**
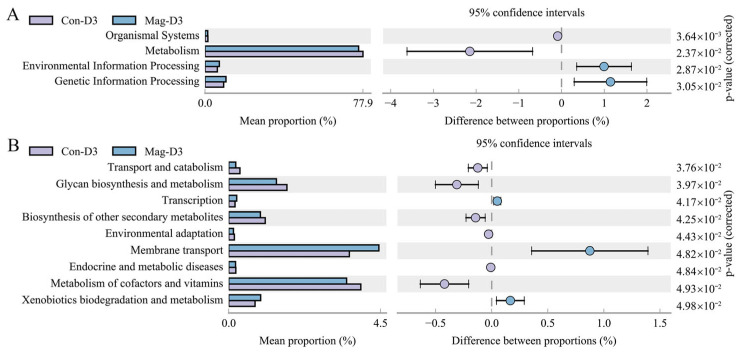
Functional differences between the Mag-treated and -untreated groups after 3 days of BEV infection. (**A**) Differential metabolic pathways among the level 1 KEGG pathways between the Con-D3 and Mag-D3 groups. (**B**) Differential metabolic pathways among the level 2 KEGG pathways between the Con-D3 and Mag-D3 groups.3.9. FMT from Magnolol-Treated Mice Inhibits the BEV Infection.

Apart from investigating the microbiota composition, exploring its functional characteristics is equally crucial. To evaluate the impact of Mag treatment on the functional and metabolic alterations in the microbiota of BEV-infected mice, we employed Phylogenetic Investigation of Communities by Reconstruction of Unobserved States (PICRUSt). Simultaneously, the functional categories were predicted using the Kyoto Encyclopedia of Genes and Genomes (KEGG) database. At 3 dpi, four KEGG pathways at level 1 showed significant differences between the Mag-treated and -untreated groups, with the altered pathways primarily concentrated in metabolism (Figure 10A). Compared with the Con-D3 group, the Mag-D3 group showed a marked increase in the relative abundance of environmental information processing and genetic information processing, while the relative abundance of organismal systems and metabolism was notably reduced. Similarly, among the level 2 KEGG pathways, nine pathways demonstrated significant differences between the Mag-treated and -untreated groups (Figure 10B). The predominant functional alterations at this classification level were concentrated in membrane transport, metabolism of cofactors and vitamins, and glycan biosynthesis and metabolism. Specifically, the Mag-D3 group exhibited a significant increase in the abundance of membrane transport and xenobiotics biodegradation and metabolism, whereas the abundance of metabolism of cofactors and vitamins, glycan biosynthesis and metabolism, and biosynthesis of other secondary metabolites was significantly decreased compared to the Con-D3 group. Additionally, the abundance of transport and catabolism, transcription, environmental adaptation, and endocrine and metabolic diseases also showed notable differences between the two groups.

**Figure 11:**
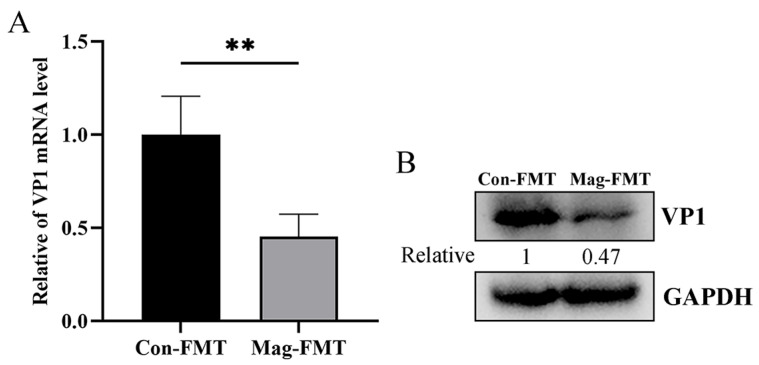
FMT from mice treated with magnolol suppresses BEV infection. (**A**) The BEV RNA level in the small intestine was determined by RT-qPCR. Unpaired *t*-test, *p* = 0.004. (**B**) The BEV VP1 protein level in the small intestine was measured by Western blot. ** *p* < 0.01.

To further confirm that magnolol inhibits BEV infection through the gut microbiota, feces samples from mice treated with or without magnolol were collected and used for FMT in BEV-infected mice. The BEV RNA and protein levels were then measured in the collected tissue specimens. Compared to the Con-FMT group, the levels of BEV RNA and protein in the small intestine of the mice receiving feces from Mag-treated donors were markedly reduced (Figure 11A,B), demonstrating that the gut microbiota was indeed involved in the suppression of BEV infection by Mag.

## 4. Discussion

Bovine enterovirus (BEV) infection is a clinically significant infectious disease caused by bovine enterovirus, which is characterized by respiratory and digestive symptoms. As the causative agent, BEV evolves through mutation and recombination and can easily mix with other pathogens, posing a serious threat to the farming industry. Therefore, exploring the pathogenic mechanism of BEV and finding effective ways to prevent and control its infection are urgent.

The gut microbiota, as a complex community, fulfills important physiological and biochemical functions in animals. Under healthy conditions, the gut microbiota maintains a dynamic balance. However, external stimuli such as dietary changes, environmental stresses, medications, and pathogens can disrupt this balance, influencing the pathogenesis of diseases. In recent years, reports have confirmed that viral infections can induce significant alterations in the gut microbiota, particularly diarrheal viruses. Zhang et al. observed that bovine viral diarrhea virus (BVDV) infection significantly altered the low-abundance bacteria in normal mice [14]. Similarly, Huang et al. reported that porcine epidemic diarrhea virus (PEDV) infection led to notable changes in the bacterial diversity, richness, and composition in piglets [37]. Moreover, Yang et al. revealed a strong correlation between rotavirus pathogenicity and variations in the intestinal microbiota [38]. In this study, we observed that BEV infection altered the gut microbial composition in mice. The abundance of *Lactobacillaceae* and *Clostridiaceae* was reduced in the BEV-infected group compared to the uninfected group, whereas *Rikenellaceae* exhibited an opposite trend. *Lactobacillus* and *Ligilactobacillus*, two vital probiotics in the microbial community, showed a continuous decline during BEV infection. Similar alterations in probiotics have also been observed in norovirus and rotavirus infection [39,40]. As a genus in the family *Rikenellaceae* (phylum *Bacteroidota*), *Alistipes* has been implicated in intestinal inflammation. A previous study reported an enrichment of *Alistipes* in fecal specimens from SAMP1/YitFc mice with Crohn’s disease (CD)-like ileitis compared to the parental ileitis-free AKR/J mice, suggesting a potential association between *Alistipes* and ileitis [41,42]. In our study, we also observed the enrichment of *Alistipes* following BEV infection. Additionally, the changes in abundance of *Candidatus_Arthromitus* and *unclassified_Rikenellaceae* were statistically significant before and after BEV infection.

Magnolol (Mag), as a polyphenolic bioactive substance, has been proven to have antiviral potential, but its effect on BEV infection remains unstudied. Therefore, we evaluated the antiviral efficacy of Mag against BEV in a BEV-infected mouse model and demonstrated that Mag effectively inhibits BEV replication in vivo. Previous studies have indicated that Mag alleviates diseases by regulating the gut microbiota in animals. Lv et al. reported that Mag ameliorates fatty liver hemorrhagic syndrome (FLHS) by modulating the gut microbiota [43]. Mo et al. found that natural magnolol reverses the gut microbiota imbalance in sick chicks infected with *Eimeria tenella* [44]. However, whether Mag can also counteract BEV-induced gut microbiota alterations to resist BEV infection remains unexplored. In this study, we performed metagenomic sequencing on the gut microbiota of BEV-infected mice in both the Mag-treated and -untreated groups. The results revealed that Mag treatment led to a reduction in the α-diversity of the microbiota compared to the untreated group, particularly at 3 dpi. Additionally, Mag treatment modulated the microbiota composition, partially reversing the BEV-induced alterations in bacterial abundance. For instance, Mag treatment significantly reversed the BEV-induced increase in *Alistipes* abundance at 3 dpi. Toomer et al. reported that the abundance of *Rikenellaceae* increased in broilers inoculated with *Salmonella enterica* Enteritidis but was restored after they were fed a feed additive supplemented with polyphenol-rich peanut skins [45]. Similarly, our study found that Mag treatment restored the *Rikenellaceae* abundance, suggesting a conserved microbial regulatory mechanism mediated by bioactive compounds. Furthermore, Mag treatment significantly reversed the BEV-induced decline in the abundance of *Lactobacillaceae* and *Lactobacillus*, demonstrating its beneficial modulatory effect on probiotics. This finding aligns with previous reports on Mag’s therapeutic potential in microbiota regulation [43,46]. To further demonstrate the role of the gut microbiota in Mag against BEV infection, we performed FMT on BEV-infected mice and found that Mag-FMT significantly protected the mice against BEV infection, which supports the feasibility of targeting the gut microbiota as an anti-BEV strategy.

In recent years, research on the interactions between viruses and the microbiota has revealed a complex bidirectional regulatory mechanism [47]. On the one hand, viral infection can disrupt the host microbiota balance, which may further enhance the viral infectivity. On the other hand, the microbiota can inhibit viral infection through various pathways. Zhang et al. found that porcine deltacoronavirus (PDCoV) infection disrupted the gut microbiota homeostasis in piglets, inducing inflammatory reactions and intestinal damage. Moreover, FMT treatment effectively regulated the intestinal microbiota composition, attenuated the inflammatory response, repaired the intestinal barrier function, and alleviated the clinical symptoms of PDCoV infection in piglets [48]. Therefore, targeted regulation of the microbiota is increasingly becoming an important strategy for combating viral infections. In the exploration of antiviral agents, significant progress has been made in probiotic research, especially *Lactobacillus* research. As a characteristically representative probiotic in the gut microbiota, *Lactobacillus* exhibits multiple protective properties, including anti-inflammatory, antiviral, and antioxidant activities [15,49,50]. Previous studies have demonstrated its potential to inhibit human enterovirus infection in vitro [51]. In this study, we observed that the dynamic changes in the *Lactobacillus* abundance during BEV pathogenesis and Mag-mediated anti-BEV defense were consistent with its antiviral characteristics, suggesting that *Lactobacillus* may serve as an important target for anti-BEV strategies in vivo. Previous studies have shown that *Lactobacillus* can exert antiviral effects through multiple pathways. For example, in a porcine rotavirus infection model, *Lactobacillus rhamnosus* GG (LGG) has been shown to improve the intestinal mucosal barrier function of piglets [52]. It has also been shown to inhibit herpes simplex virus type 2 replication by regulating IFN-I through the RIG-I signaling pathway [53]. In porcine epidemic diarrhea virus (PEDV) infection, LGG not only alleviates intestinal damage in piglets but also ameliorates lipid metabolism disorders and inflammation while improving the antioxidant capacity [54]. Additionally, acetic acid generated by *Lactobacillus* metabolism effectively mitigates PEDV infection in piglets by modulating the immune function and intestinal barrier integrity [55]. These findings underscore the need for further research into the anti-BEV role of *Lactobacillus* and its underlying mechanisms.

It is well established that alterations in the microbiota composition may be accompanied by corresponding changes in the microbial function. In this study, we employed PICRUSt to predict the gene functions of the gut microbiota and subsequently mapped the predicted functions onto specific metabolic pathways using the KEGG database, thereby elucidating the variations in the metabolic potential of microbial communities across the experimental groups. The results revealed that Mag treatment significantly modulated the metabolic pathways of the microbiota among both level 1 and level 2 KEGG functional categories in BEV-infected mice. Notably, these functional changes were predominantly concentrated in metabolism-related pathways, indicating that the gut microbiota may participate in the regulation of viral infection through metabolic mechanisms [56,57].

Our study revealed the association between magnolol, the gut microbiota, and BEV infection based on a mouse model, which laid the foundation for the prevention and control of BEV infection. Firstly, magnolol, a natural plant extract, has been shown to be a potential feed additive in the farming industry. For instance, as a feed additive, magnolol can significantly ameliorate the intestinal mucosal condition and antioxidant capacity of *Linwu* ducks, thereby enhancing their growth performance [58]. The addition of magnolol solid dispersion can not only improve the growth performance and antioxidant capacity of calves but also optimize the composition of calves’ intestinal microbiota by increasing the abundance of beneficial bacteria and reducing the abundance of harmful bacteria [59]. The combination of antiviral and growth-promoting properties gives magnolol a unique advantage for BEV prevention and control in cattle. Secondly, although there are differences in the composition of the gut microbiota between cattle and mice, the functional conservation among bacteria provides a biological basis for transformation research, especially the function of probiotics. Multiple studies have confirmed the effectiveness of probiotic intervention in the prevention and control of bovine viral diseases. Probiotics such as *Limosilactobacillus fermentum* can ameliorate the microbiota disturbance caused by rotavirus in calves [60], while microencapsulated *Lactobacillus acidophilus* NCDC15 may serve as a safe and effective adjuvant therapy for the treatment of acute rotavirus enteritis in newborn calves [61]. Therefore, the association between the gut microbiota and BEV infection identified in this study may have similar implications in cattle, and targeted regulation of the specific microbiota may establish a microecological barrier against BEV infection. In addition, this study confirmed the important role of the gut microbiota in magnolol-mediated resistance to BEV infection through FMT. Notably, FMT has been applied to treat diseases in cattle [62], which further suggests that targeted regulation of the gut microbiota (such as supplementing probiotics or conducting microbiota transplantation) may become a new strategy for preventing and controlling BEV infection in cattle.

While our findings have positive implications for combating BEV infection, this study still has certain limitations. First, the antiviral effects were evaluated using a mouse model. Given that cattle are the natural hosts of BEV, further validation of the antiviral efficacy in cattle is necessary. Second, as a preliminary exploratory study on preventing and controlling BEV infection, this work is of significant value. However, the limited number of mice in the current experiments makes it necessary for future studies to expand the sample size for more in-depth validation. Finally, it remains unclear whether the changes in the gut microbiota induced by Mag treatment directly modulate BEV replication or mediate the antiviral effects through the regulation of the host immune response, both of which require further investigation. In summary, this study demonstrates that Mag suppresses BEV replication in vivo and highlights the important role of the gut microbiota in its anti-BEV mechanism. These findings establish Mag as a potential candidate drug for combating BEV infection while laying the groundwork for investigating the anti-BEV effects of specific bacteria, such as *Lactobacillus*, and offering novel strategies for BEV prevention and control.

## Figures and Tables

**Table 1:** Primers used for qPCR.

Gene Name	Forward Primer Sequence (5′-3′)	Reverse Primer Sequence (5′-3′)
HY12-VP1	CCACTGATGCAACACCCGCTCTA	CGCTTGTTTCATGTATGCCGTGTG
m-GAPDH	AGGTCGGTGTGAACGGATTTG	GGGGTCGTTGATGGCAACA

m represents mouse.

## Data Availability

All data created in this study are available.

## References

[1] Chen J., Meng W., Zeng H., Wang J., Liu S., Jiang Q., Chen Z., Ma Z., Wang Z., Li S. (2024). Epidemiological survey of calf diarrhea related viruses in several areas of Guangdong Province. Front. Microbiol..

[2] Simmonds P., Adriaenssens E.M., Lefkowitz E.J., Oksanen H.M., Siddell S.G., Zerbini F.M., Alfenas-Zerbini P., Aylward F.O., Dempsey D.M., Dutilh B.E. (2024). Changes to virus taxonomy and the ICTV Statutes ratified by the International Committee on Taxonomy of Viruses (2024). Arch. Virol..

[3] Moll T., Davis A.D. (1959). Isolation and characterization of cytopathogenic enteroviruses from cattle with respiratory disease. Am. J. Vet. Res..

[4] Blas-Machado U., Saliki J.T., Boileau M.J., Goens S.D., Caseltine S.L., Duffy J.C., Welsh R.D. (2007). Fatal ulcerative and hemorrhagic typhlocolitis in a pregnant heifer associated with natural bovine enterovirus type-1 infection. Vet. Pathol..

[5] Shaukat S., Angez M., Alam M.M., Sharif S., Khurshid A., Malik F., Rana M.S., Mahmood T., Zaidi S.S. (2012). Molecular identification and characterization of a new type of bovine enterovirus. Appl. Environ. Microbiol..

[6] Zhu L., Xing Z., Gai X., Li S., San Z., Wang X. (2014). Identification of a novel enterovirus E isolates HY12 from cattle with severe respiratory and enteric diseases. PLoS ONE.

[7] Sobhy N.M., Mor S.K., Mohammed M.E., Bastawecy I.M., Fakhry H.M., Youssef C.R., Abouzeid N.Z., Goyal S.M. (2015). Isolation and molecular characterization of bovine enteroviruses in Egypt. Vet. J..

[8] Kosoltanapiwat N., Yindee M., Chavez I.F., Leaungwutiwong P., Adisakwattana P., Singhasivanon P., Thawornkuno C., Thippornchai N., Rungruengkitkun A., Soontorn J. (2016). Genetic variations in regions of bovine and bovine-like enteroviral 5′UTR from cattle, Indian bison and goat feces. Virol. J..

[9] He H., Tang C., Chen X., Yue H., Ren Y., Liu Y., Zhang B. (2017). Isolation and characterization of a new enterovirus F in yak feces in the Qinghai-Tibetan Plateau. Arch. Virol..

[10] Karim A. (2024). Unveiling the Potential of Probiotics in Osteoarthritis Management. Curr. Rheumatol. Rep..

[11] Qin M., Huang Z., Huang Y., Huang X., Chen C., Wu Y., Wang Z., He F., Tang B., Long C. (2024). Association analysis of gut microbiota with LDL-C metabolism and microbial pathogenicity in colorectal cancer patients. Lipids Health Dis..

[12] Du L., Ding X., Tian Y., Chen J., Li W. (2024). Effect of anthocyanins on metabolic syndrome through interacting with gut microbiota. Pharmacol. Res..

[13] Zhang Y., Wang H., Sang Y., Liu M., Wang Q., Yang H., Li X. (2024). Gut microbiota in health and disease: Advances and future prospects. MedComm.

[14] Zhang Z., Huang J., Li C., Zhao Z., Cui Y., Yuan X., Wang X., Liu Y., Zhou Y., Zhu Z. (2024). The gut microbiota contributes to the infection of bovine viral diarrhea virus in mice. J. Virol..

[15] Cao Z., Ling X., Sun P., Zheng X., Zhang H., Zhong J., Yin W., Fan K., Sun Y., Li H. (2023). Matrine Targets Intestinal Lactobacillus acidophilus to Inhibit Porcine Circovirus Type 2 Infection in Mice. Int. J. Mol. Sci..

[16] Zhao Z., Li C., Huang J., Yuan X., Cui Y., Liu Y., Zhou Y., Zhu Z., Zhang Z. (2024). Phlorizin Limits Bovine Viral Diarrhea Virus Infection in Mice via Regulating Gut Microbiota Composition. J. Agric. Food Chem..

[17] Dou B., Wu X., He Y., Xu G., Zhang H., Huang Q., Chen X., Duan N., Zhou L., Zhang W. (2024). Fei-Yan-Qing-Hua decoction attenuates influenza virus infection by enhancing host antiviral response through microbiota-derived acetate. Front. Pharmacol..

[18] Pan W., Wu R., Zhang Q., Ma Y., Xiang J., Wang J., Chen J. (2024). Ruhao Dashi granules exert therapeutic effects on H1N1 influenza virus infection by altering intestinal microflora composition. Front. Microbiol..

[19] Jiang J., Hou X., Xu K., Ji K., Ji Z., Xi J., Wang X. (2024). Bacteria-targeted magnolol-loaded multifunctional nanocomplexes for antibacterial and anti-inflammatory treatment. Biomed. Mater..

[20] Tseng C.F., Chen H.M., Liao T.L., Hsu F.T., Yeh C.J., Chen W.T., Kok S.H. (2024). Magnolol’s Therapeutic Efficacy and Immunomodulatory Effects in Oral Squamous Cell Carcinoma. In Vivo.

[21] Peng W.S., Gao M., Yao X.F., Tong Y.Y., Zhang H.H., He X. (2023). Magnolol supplementation alleviates diquat-induced oxidative stress via PI3K-Akt in broiler chickens. Anim. Sci. J..

[22] Zhao D., Guo X., Lin B., Huang R., Li H., Wang Q., Zeng Y., Shang Y., Wu Y. (2024). Magnolol against enterovirus 71 by targeting Nrf2-SLC7A11-GSH pathway. Biomed. Pharmacother..

[23] Wang X., Chen B., Yu R., Si F., Xie C., Li Z., Dong S., Zhang D. (2023). Magnolol, a Neolignan-like Drug, Inhibits Porcine Epidemic Diarrhea Virus Replication in Cultured Cells. Pathogens.

[24] Kim H., Lim C.Y., Chung M.S. (2021). Magnolia officinalis and Its Honokiol and Magnolol Constituents Inhibit Human Norovirus Surrogates. Foodborne Pathog. Dis..

[25] Chen X., Hao K., Yu X., Huang A., Zhu B., Wang G.X., Ling F. (2018). Magnolol protects Ctenopharyngodon idella kidney cells from apoptosis induced by grass carp reovirus. Fish Shellfish Immunol..

[26] Li J., Huang Y., Guan X.L., Li J., Deng S.P., Wu Q., Zhang Y.J., Su X.J., Yang R.Y. (2012). Anti-hepatitis B virus constituents from the stem bark of Streblus asper. Phytochemistry.

[27] Li X. (2019). Effect of IRF3/7-Mediated Innate Immunity on HY12 Enterovirus Replication. Master’s Thesis.

[28] Wang G. (2021). Isolation and Identification of Bovine Enterovirus and Development of Bivalent Inactivated Vaccine. Master’s Thesis.

[29] Tao W., Hu Y., Chen Z., Dai Y., Hu Y., Qi M. (2021). Magnolol attenuates depressive-like behaviors by polarizing microglia towards the M2 phenotype through the regulation of Nrf2/HO-1/NLRP3 signaling pathway. Phytomedicine.

[30] Bai Y., Song L., Dai G., Xu M., Zhu L., Zhang W., Jing W., Ju W. (2018). Antidepressant effects of magnolol in a mouse model of depression induced by chronic corticosterone injection. Steroids.

[31] Matsui N., Akae H., Hirashima N., Kido Y., Tanabe S., Koseki M., Fukuyama Y., Akagi M. (2016). Magnolol Enhances Hippocampal Neurogenesis and Exerts Antidepressant-like Effects in Olfactory Bulbectomized Mice. Phytother. Res..

[32] Yang J., Wei Y., Zhao T., Li X., Zhao X., Ouyang X., Zhou L., Zhan X., Qian M., Wang J. (2022). Magnolol effectively ameliorates diabetic peripheral neuropathy in mice. Phytomedicine.

[33] Xu C., Ye J., Sun Y., Sun X., Liu J.G. (2024). The Antidepressant Effect of Magnolol on Depression-like Behavior of CORT-Treated Mice. J. Mol. Neurosci..

[34] Xia T., Zhang J., Han L., Jin Z., Wang J., Li X., Man S., Liu C., Gao W. (2019). Protective effect of magnolol on oxaliplatin-induced intestinal injury in mice. Phytother. Res..

[35] Chang X., Guo Y., Zhang Q., Zheng X., Cui X., Hu J., Zhang Z., Zhang F., Wang X. (2024). GRP78 recognizes EV-F 3D protein and activates NF-kappaB to repress virus replication by interacting with CHUK/IKBKB. J. Virol..

[36] Fluhr L., Mor U., Kolodziejczyk A.A., Dori-Bachash M., Leshem A., Itav S., Cohen Y., Suez J., Zmora N., Moresi C. (2021). Gut microbiota modulates weight gain in mice after discontinued smoke exposure. Nature.

[37] Huang A., Cai R., Wang Q., Shi L., Li C., Yan H. (2019). Dynamic Change of Gut Microbiota During Porcine Epidemic Diarrhea Virus Infection in Suckling Piglets. Front. Microbiol..

[38] Yang H., Fan X., Mao X., Yu B., He J., Yan H., Wang J. (2024). The protective role of prebiotics and probiotics on diarrhea and gut damage in the rotavirus-infected piglets. J. Anim. Sci. Biotechnol..

[39] Lee H., Ko G. (2017). New perspectives regarding the antiviral effect of vitamin A on norovirus using modulation of gut microbiota. Gut Microbes.

[40] Zhao W., Yu M.L., Tao X., Cheng M.H., Liu C.C., Liu Y., Li Y.G. (2021). Analysis of the intestinal microbial community altered during rotavirus infection in suckling mice. Virol. J..

[41] Parker B.J., Wearsch P.A., Veloo A.C.M., Rodriguez-Palacios A. (2020). The Genus Alistipes: Gut Bacteria With Emerging Implications to Inflammation, Cancer, and Mental Health. Front. Immunol..

[42] Rodriguez-Palacios A., Harding A., Menghini P., Himmelman C., Retuerto M., Nickerson K.P., Lam M., Croniger C.M., McLean M.H., Durum S.K. (2018). The Artificial Sweetener Splenda Promotes Gut Proteobacteria, Dysbiosis, and Myeloperoxidase Reactivity in Crohn’s Disease-like Ileitis. Inflamm. Bowel Dis..

[43] Lv Y., Ge C., Wu L., Hu Z., Luo X., Huang W., Zhan S., Shen X., Yu D., Liu B. (2024). Hepatoprotective effects of magnolol in fatty liver hemorrhagic syndrome hens through shaping gut microbiota and tryptophan metabolic profile. J. Anim. Sci. Biotechnol..

[44] Mo J., Xiang J., Li J., Yang M., Zhang Z., Zhang L., Zhang G., Yang Y., Liu G., Lu Y. (2023). Natural Magnolol ameliorates coccidiosis infected with Eimeria tenella by affecting antioxidant, anti-inflammatory, and gut microbiota of chicks. Poult. Sci..

[45] Toomer O.T., Redhead A.K., Vu T.C., Santos F., Malheiros R., Proszkowiec-Weglarz M. (2024). The effect of peanut skins as a natural antimicrobial feed additive on ileal and cecal microbiota in broiler chickens inoculated with Salmonella enterica Enteritidis. Poult. Sci..

[46] Chen F., Zhang H., Du E., Fan Q., Zhao N., Jin F., Zhang W., Guo W., Huang S., Wei J. (2021). Supplemental magnolol or honokiol attenuates adverse effects in broilers infected with Salmonella pullorum by modulating mucosal gene expression and the gut microbiota. J. Anim. Sci. Biotechnol..

[47] Li N., Ma W.T., Pang M., Fan Q.L., Hua J.L. (2019). The Commensal Microbiota and Viral Infection: A Comprehensive Review. Front. Immunol..

[48] Zhang Y., Si L., Shu X., Qiu C., Wan X., Li H., Ma S., Jin X., Wei Z., Hu H. (2025). Gut microbiota contributes to protection against porcine deltacoronavirus infection in piglets by modulating intestinal barrier and microbiome. Microbiome.

[49] Zang R., Zhou R., Li Y., Wu H., Lu L., Xu H. (2024). The probiotic Lactobacillus plantarum alleviates colitis by modulating gut microflora to activate PPARgamma and inhibit MAPKs/NF-kappaB. Eur. J. Nutr..

[50] Liu W., Cheng H., Zhang H., Liu G., Yin X., Zhang C., Jiang R., Wang Z., Ding X. (2024). Effect of Lactobacillus paracasei LK01 on Growth Performance, Antioxidant Capacity, Immunity, Intestinal Health, and Serum Biochemical Indices in Broilers. Animals.

[51] Ang L.Y., Too H.K., Tan E.L., Chow T.K., Shek L.P., Tham E.H., Alonso S. (2016). Antiviral activity of Lactobacillus reuteri Protectis against Coxsackievirus A and Enterovirus 71 infection in human skeletal muscle and colon cell lines. Virol. J..

[52] Mao X., Gu C., Hu H., Tang J., Chen D., Yu B., He J., Yu J., Luo J., Tian G. (2016). Dietary *Lactobacillus rhamnosus* GG Supplementation Improves the Mucosal Barrier Function in the Intestine of Weaned Piglets Challenged by Porcine Rotavirus. PLoS ONE.

[53] Wang J., Huang M., Du Y., Chen H., Li Z., Zhai T., Ou Z., Huang Y., Bu F., Zhen H. (2024). *Lactobacillus rhamnosus* GG Regulates Host IFN-I Through the RIG-I Signalling Pathway to Inhibit Herpes Simplex Virus Type 2 Infection. Probiotics Antimicrob. Proteins.

[54] Xu Z., Zhang Q., Wu M., Zhang Y., Li Z., Li H., Yu C., Zhang X., Zhao D., Wang L. (2024). *Lactobacillus rhamnosus* GG powder supplementation alleviates intestinal injury in piglets challenged by porcine epidemic diarrhea virus. Front. Cell. Infect. Microbiol..

[55] Sun M.J., Xing J.H., Yan Q.S., Zou B.S., Wang Y.J., Niu T.M., Yu T., Huang H.B., Zhang D., Zhang S.M. (2024). The Acetic Acid Produced by Lactobacillus Species Regulates Immune Function to Alleviate PEDV Infection in Piglets. Probiotics Antimicrob. Proteins.

[56] Nelson C.A., Wilen C.B., Dai Y.N., Orchard R.C., Kim A.S., Stegeman R.A., Hsieh L.L., Smith T.J., Virgin H.W., Fremont D.H. (2018). Structural basis for murine norovirus engagement of bile acids and the CD300lf receptor. Proc. Natl. Acad. Sci. USA.

[57] Li W., Chen H., Tang J. (2024). Interplay between Bile Acids and Intestinal Microbiota: Regulatory Mechanisms and Therapeutic Potential for Infections. Pathogens.

[58] Lin Q., Zhao J., Xie K., Wang Y., Hu G., Jiang G., Dai Q., Fan Z., He J., He X. (2017). Magnolol additive as a replacer of antibiotic enhances the growth performance of Linwu ducks. Anim. Nutr..

[59] Zhang Y., Wang Q., Shi X., Guo Z., He X., Zhang T., Zhao X. (2025). Effects of magnolol solid dispersion on growth performance, serum antioxidant capacity and intestinal microbiome of calves. Acta Vet. et Zootech. Sin..

[60] Murtaza N., Nawaz M., Yaqub T., Mehmood A.K. (2024). Impact of Limosilactobacillus fermentum probiotic treatment on gut microbiota composition in sahiwal calves with rotavirus diarrhea: A 16S metagenomic analysis study. BMC Microbiol..

[61] Gandhar J.S., De U.K., Kala A., Malik Y.S., Yadav S., Paul B.R., Dixit S.K., Sircar S., Chaudhary P., Patra M.K. (2022). Efficacy of Microencapsulated Probiotic as Adjunct Therapy on Resolution of Diarrhea, Copper-Zinc Homeostasis, Immunoglobulins, and Inflammatory Markers in Serum of Spontaneous Rotavirus-Infected Diarrhoetic Calves. Probiotics Antimicrob. Proteins.

[62] Kim H.S., Whon T.W., Sung H., Jeong Y.S., Jung E.S., Shin N.R., Hyun D.W., Kim P.S., Lee J.Y., Lee C.H. (2021). Longitudinal evaluation of fecal microbiota transplantation for ameliorating calf diarrhea and improving growth performance. Nat. Commun..

